# Hearing status in patients with rheumatoid arthritis

**DOI:** 10.22088/cjim.10.4.447

**Published:** 2019

**Authors:** Keyvan Kiakojuri, Behnaz Yousef Ghahari, Sanaz Soltanparast, Mohsen Monadi

**Affiliations:** 1 Department of ENT Babol University of Medical Sciences, Babol, Iran; 2 Department of Internal Medicine, Babol University of Medical Sciences, Babol, Iran; 3 Department of Audiology, School of Rehabilitation, Iran University of Medical Sciences, Tehran, Iran

**Keywords:** Rheumatoid arthritis, Sensorineural hearing loss, Audiometry, Tympanometry, Speech perception, Hearing loss

## Abstract

**Background:**

Previous studies showed that one of the complications of rheumatoid arthritis disease was auditory disorder. The goal of the present study was to compare the auditory status in patients with rheumatoid arthritis and healthy individuals.

**Methods:**

In the present case-control study, 30 normal persons and 60 persons with rheumatoid arthritis with mean age of 46.72 and standard deviation of 6.76 of both genders were appraised using pure tone audiometry, tympanometry and speech audiometry. The mean disease duration in patients with rheumatoid arthritis was 12.51±6.09 years.

**Results:**

The frequency of hearing loss in rheumatoid arthritis group was significantly more than the control group (p=0.001). All patients had sensorineural hearing loss. Only in 5% of rheumatoid arthritis group, abnormal tympanometry (as type) was reported. Speech discrimination score analysis showed significant difference between the patients with rheumatoid arthritis and controls. In terms of hearing threshold level, the mean hearing threshold level (in 2000, 4000 and 8000 Hz frequencies) of the patients with rheumatoid arthritis was significantly higher than control group in both ears (p<0.05). A positive significant correlation was found among mean hearing threshold level in 4000 and 8000 Hz frequencies and rheumatoid arthritis duration in both ears.

**Conclusion:**

The frequency of hearing loss and the average hearing threshold in RA patients were higher than healthy individuals. The most common type hearing loss is sensorineural.

Rheumatoid arthritis (RA) is a systemic long-lasting inflaming illness that impacts roughly 1% of the global people and is specified by fervor of the synovial coats of diarthrodial joints, which result in oncoming demolition of articular and periarticular textures (1-3). It is accompanied with oncoming inability, systemic difficulties, timely death and socioeconomic expenses(4, 5). RA may entail alternative body systems containing heart, lung, skin, and eye (6, 7). In this regard, the possibility of auditory system contention in RA has been one of the domains with great concern. Raut et al. (2001) contrasted 35 patients with RA with 35 age- and sex-matched controls to assess the relation between RA and hearing disability (8). The finding revealed a considerable hearing disability at 500 Hz, 1.0 kHz, and 2.0 kHz in patients with RA (8). Ozcan et al. (2002) completed a controlled study to investigate the outbreak of hearing loss in RA (9). Their study recorded that the outbreak of hearing impairment was considerably higher in patients with RA(9). Halligan et al. (2006) performed a case‐control cross‐sectional study of 29 patients with RA with illness period more than 5 years to measure if patients with rheumatoid arthritis (RA) are more presumably to possess subclinical hearing loss contrasted to individuals deprived of RA (10).

According to the results, individuals with RA did not display any objective hearing threshold dissimilarity from subjects deprived of RA (10). However, in general the auditory system is negatively affected in patients with RA, but the specifications of the hearing impairment (HI) are insignificantly realized until now. Both sensorineural hearing loss (SNHL), conductive hearing loss (CHL) and or mixed type have been recorded. Currently, the pathogenesis of hearing disability in RA is not distinctly recognized (11). Nevertheless, the incudostapedial and incudomalleolar joints involvement by the similar inflammatory and ruinous modifications as another joint, could make CHL and the inner ear lesion because of vasculitis or neuritis, or the ototoxicity of antirheumatic drugs used for RA treatment leading to SNHL(12-14). 

Since the relationship among RA and hearing impairment is as again argumentative(1, 15, 16) and just a little systematic inquiries have contrasted the proficiency and feasible impairment of the auditory system in patients with RA, so the goal of present study was to specify and compare hearing impairment in these group of patients and healthy controls employing pure tone audiometry (PTA), speech audiometry and tympanometry. 

On the other hand, in this study with the establishment of age limits, the elimination of presbycusis was achieved for the first time in Iran. 

## Methods

In this study, based on previous similar study, 30 normal individuals and 60 patients with RA were evaluated. Normal individuals and patients with RA were selected from cases attending a rheumatology clinic at Ayatollah Rouhani Hospital, Babol.

The patients with RA were not included in the study if there was any history of hearing aid use, familial hearing loss, neurological problems, congenital head and neck anomalies, convulsions, epilepsy, head trauma, ototoxic medications, brain surgery and RA more than 5 years according to medical records. In an age-matched control group, besides the mentioned items, absence of RA was also confirmed by a skilled rheumatologist. All patients and volunteers gave informed consent and this research was conducted with the approval of the Ethics Committee of Babol University of Medical Sciences. First, a comprehensive case history was gathered by an expert researcher via a definite questionnaire, covered the demographic, socioeconomic and clinical specifications of the study population, in the Otolaryngology Clinic of Ayatollah Rouhani Hospital. Then a thorough ENT inspection was done for every contributor with specific stress on hearing discontents. Otoscopic inspection was performed to examine the ear canal, cerumen, and healthy eardrum at the Otolaryngology Clinic of Ayatollah Rohani Hospital. If there was a cerumen, cleansing was done. Then audiological valuation was carried out in a sound-proof room and included evaluation of pure tone audiometry, speech audiometry and tympanometry was applied to all patients and controls via skilled audiologist who was sightless to the study group contributor. 

Pure tone audiometry was performed to check each person’s hearing status at frequencies of 250,500, 1000, 2000, 4000 and 8000 Hz using an ASTRA audiometer and TDH39 headphone. Hearing was accepted as abnormal if the hearing threshold was higher than 20 dB at each frequency. Speech audiometry included SRT and SDS using dissyllabic and monosyllabic words respectively, was also performed/evaluated. 

Tympanometric tests were performed to determine tympano-ossicular chain compliance and middle-ear pressure with a Zodiac (Madsen) impedance audiometer at a probe tone frequency of 226 Hz. Statistical analysis was conducted by SPSS 18 statistical software at significance level of p<0.05. Since the data distribution was normal, Pearson correlation coefficient, one way ANOVA, t-test and chi square were used for statistical analysis. 

## Results

Our study was conducted on 60 patients with RA (55 females and 5 males) with a mean age of 47.20 and standard deviation of 7.43 and 30 normal individuals (24 females and 6 males) with mean age of 45. 76 and standard deviation of 5.17 years, aged between 18-60 years**.**

The basic information of the control group and the group of people with RD showed frequency of hearing loss in rheumatoid arthritis group was significantly more than the control group (p=0.001) and all of whom showed a SNHL. The mean threshold value for both ears of the patients with RA and the controls were compared (table 1). 

A statistically significant difference was found between the average of hearing thresholds of both ears in the two groups.

**Table 1:** Comparison of the mean threshold value in patients with RA and the controls in the right and left ears

**Frequency**	**RA** **(mean±SD)**	**Control** **(mean±SD)**	**P value**
250 500 1000 2000 4000 8000	7.40±3.67 8.70±3.47 10.30±3.69 14.1±7.86 16.9±11.24 23±16.51	6.33±3.92 7.33±3.40 9.0±3.05 11.16±3.95 11.1±4.08 13.3±6.47	0.22 0.09 0.1 0.002 0.002 0.001

In 3 (5%) patients of the RA group, an As-type tympanogram (Reduced mobility of the tympanic membrane caused by a stiffened middle ear system can cause a shallow peak on the tympanogram, called a Type A_s_ tympanogram) was observed, 2 of whom showed bilateral As type, whereas 1 patient showed a unilateral as type. All persons in the control group showed normal tympanogram. As shown in table 2, a positive significant correlation was found among disease duration and mean hearing threshold value in both ears at 4000Hz and 8000Hz.

**Table 2:** Assessing the correlation among disease duration and mean hearing threshold value in both ears

Hearing threshold							RA duration
**8000**	**4000**	**2000**	**1000**	**500**	**250**		
0.300 0.03*	0.349 0.01*	0.204 0.15	0.203 0.15	0.272 0.06	0.182 0.20	r Pvalue	Right ear
0.294 0.03*	0.295 0.03*	0.256 0.07	0.218 0.12	0.096 0.50	0.174 0.22	r Pvalue	Left ear

RA (rheumatoid arthritis)

Table 3 shows the mean and standard deviation of SDS and SRT values for both ears of the control group and the group of people with RD. These results revealed no statistically significant difference between the average of SRT values of both ears of persons in RA and control groups. While a statistically significant difference was noted for mean SDS values in both ears between the patients with RA and controls.

**Table 3:** Descriptive statistics of SDS and SRT values in control group and the group of people with RD

**P-value**	**Control**	**RA**	**Speech audiometry**	**Variable**
0.08 0.03	10.34±3.26 96.07±3.29	14.28±5.83 93.56±4.69	SRT SDS	Right ear
0.17 0.04	10.63±3.6595.84±3.62	13.02±6.1393.28±3.46	SRT SDS	Left ear

SRT (speech recognition threshold) SDS (speech discrimination score)

RA (rheumatoid arthritis)

## Discussion

This study has shown a higher spread of hearing loss, particularly sensorineural kind, in RA patients. Our data were in line with those of the prior search that demonstrated subjects with RA have more presumably possess a SNHL(13, 17-24).nonetheless, Rosenberg et al. (1978) could not detect any degree of relation among RA and hearing loss(25). In determining the correlation between RA disease duration and hearing impairments, a positive significant correlation was found among disease duration and mean hearing threshold value in both ears at 4000Hz and 8000Hz. Ozturk et al. (2004) and Dikici et al. (2009) studies also concurred with the detection of the present study and demonstrated that hearing impairment progressed with the duration of the disease (11, 13). In 2011, Arslan et al. examined the relation between incidence of hearing loss and duration of disease in 44 RA patients with mean age of 47.2±11.2(26). The results of this study showed no relation between the incidence of hearing loss and duration of disease(26). In 2016, Vega et al. investigated hearing in RA population(12). They carried out their study on fifty-three patients with rheumatoid arthritis and 71 patients with an indigenous condition of health who were matched for age and gender(12). In their study, no correlation was found between those diagnosed with SNHL using PTA and the duration of the disease(12). These inconsistencies may be due to the differences in the characteristics of studied subjects. 

We showed the type A tympanogram only in 5% of patients. Our results are similar to Gonzalez et al. (2015) , Lobo et al. (2016) , Rahne et al. (2017) , Galarza-Delgado et al. (2017) and Ahmadzade et al.’s (2017) studies which demonstrated no significant difference between the stiffness of ossicular chains and RA(15, 27-30) .In 2011, Arslan et al. compared 44 RA patients with 44 voluntary healthy controls(26). The results of this study showed that there were significant differences among the two studied groups in terms of Type A tympanogram (26). Takatsu et al. (2005) evaluated the degree of hearing impairment in patients with rheumatoid arthritis (RA) (2). The findings of this study showed the type- A tympanogram significantly increased in the patients with RA compared with the controls (2). Ozcan et al. (2002) investigated hearing and middle ear functions in 37 patients with RA and 35 controls to study the prevalence and the nature of hearing loss in RA (9). The results of this study showed higher prevalence of abnormal tympanogram in RA patients (9). In 1980, Reiter et al. measured the middle ear immittance in RA patients (31). In their study, immittance data disclosed abnormal discoveries in 59% of the patients (31). The discrepancies between these findings may be due to differences in the mean age and the number of samples. In the present study, the relation between speech reception threshold and RA and the findings showed that this test has failed to separate patients with RA from controls. On the other hand, in this study a statistically significant difference was noted for mean SDS values in both ears between the patients with RA and controls. So it can be supposed that the disease process touched beyond the cochlea. Unlike the present study, Ozcan et al. , Raut et al. and Ahmadzade et al.’s study results showed no statistically significant difference between the two groups in terms of mean SDS values (9, 20, 28). According to Galarza-Delgado et al.’s studies in 2017, RA subjects recognized >70% of the monosyllabic and trisyllabic words in speech audiometry (29). More studies are needed to clear the difference. For future researches, we recommend that the patients' auditory status should be compared in active and passive phases of the disease.

In conclusion the present study showed that the frequency of hearing loss in rheumatoid arthritis group was significantly more than the control group**. **Accordingly, audiological assessment should be considered in routine evaluation of patients with RA, to prevent hearing-related handicap. 
